# Relationship between vertical facial patterns and dental arch form in class II malocclusion

**DOI:** 10.1186/2196-1042-14-43

**Published:** 2013-11-07

**Authors:** Cristina Grippaudo, Bruno Oliva, Anna Lucia Greco, Simone Sferra, Roberto Deli

**Affiliations:** Catholic University of Sacred Heart, Largo Francesco Vito 1, Rome, 00198 Italy

**Keywords:** Arch form, Class II malocclusion, Vertical facial pattern, Software

## Abstract

**Background:**

The purpose of this study is to evaluate the relationship between dental arch form and the vertical facial pattern determined by the angle between the mandibular plane and the anterior cranial base (Sella-nasion/mandibular plane angle (SN-MP)) in skeletal class II untreated patients.

**Methods:**

A sample of 73 Caucasians patients with untreated skeletal class II in permanent dentition was divided into three groups according to the values of the angle SN-MP. An evaluation of the arch form was performed by angular and linear relation values on each patient. Regression analysis was used to determine the statistical significance of the relationships between SN-MP angle and dental arch form. The differences among the three groups were analyzed for significance using a variance analysis.

**Results:**

A decrease of the upper arch transversal diameters in high SN-MP angle patients and an increase in low angle SN-MP ones (*P* < 0.05) were shown. Result analysis showed a change in upper arch shape, with a smaller intercanine width in patients with high SN-MP angle and a greater one in low angle patients. As SN-MP angle increased, the upper arch form tended to be narrower. No statistically significant difference in mandibular arch form among the three groups was found, except the angle value related to incisors position.

**Conclusions:**

The results showed the association between the upper dental arch form and the vertical facial pattern. On the contrary, the lower arch form was not related to the mandibular divergence.

## Background

The determination of dental arch forms is a multifactorial trait. The genetic component could be partly related to vertical growth patterns and to environmental components related to functional, muscular, and local factors [1]. Orthodontic treatments are conditioned by arch forms, which must be respected to avoid serious consequences, such as relapse or iatrogenic damage to teeth being moved beyond their bone edges. Orthodontic arch wires are manufactured in several different forms of dental arch in order to give the orthodontist the chance to choose the most suitable ones for each patient. Several authors aimed their research in order to find out the ideal arch form [2–4]. Therefore, there are many different arch forms that orthodontic manufacturer produce as archwires, and it is difficult to choose the most suitable for our patients. A research that analyzed the arch form of the Italian population found that none of the commercial archwire fits exactly the patient archform [5].

The purpose of the present study is to investigate the relationship between dental arch forms and vertical growth patterns in skeletal class II malocclusions.

The shape of the tooth arch is related to the vertical dimension as well as the jaw transverse is related to the vertical skeletal growth.

Isaacson et al*.*[6] reported that subjects with long faces showed decreased maxillary intermolar width. Nasby et al. [7] noted increased mandibular molar diameters and length of maxillary and mandibular arches in subjects with reduced Sella-nasion/mandibular plane angle (SN-MP). Forster et al. [8] showed that the transverse diameters were reduced in both males and females with high-angle SN-MP.

Taking the anterior cranial base (SN) as a reference point to determine the inclination of the mandibular plane (MP) according to Schudy [9], patients can be differentiated as individuals with high-angle SN-MP and long face and as individuals with low-angle SN-MP and short face [10, 11]*.* The jaw transverse dimensions are also related to the vertical growth patterns. Long-face individuals have small skeletal transversal dimensions and individuals featuring short face have increased cross-sectional dimensions [12].

The knowledge of the relationship between dental and skeletal characteristics helps both in diagnostic assessment and in treatment planning.

## Methods

### Sample

A sample of 73 untreated Caucasian subjects with skeletal class II malocclusion (A point, Nasion, B point (ANB) average = 6.2°), aged between 11 and 38 years, was included in this study. Inclusion criteria were permanent dentition except third molars, pre-treatment lateral cephalogram, dental casts, and photographs. Exclusion criteria were malformations, edentulous spaces, and previous orthodontic treatment.

The sample, for descriptive purposes, was divided into three groups according to the values of the angle SN-MP:


These values represent one standard deviation (SD) from the average SN-MP angle reported by the Italian Board of Orthodontics (IBO) and European Board of Orthodontics (EBO).

A preliminary analysis of sample size revealed that the number of subjects enrolled in the three groups under investigation warranted a power of the study of 0.80 and an *α* of 0.05 on the basis of the standard deviation (1°) of a clinically relevant value (3°). These values were performed on three commercial dental archforms. The Shapiro-Wilk test revealed normality of distribution of the data.

### Measurements

Lateral head cephalogram and photographs of pre-treatment plaster study models were measured. For each subject, SN-MP angle was measured.

The shape of dental arches was measured on digital photographs of patient plaster models. The evaluation of the dental arch form was performed on angular measurements and linear relationships using a computer analysis.

**Figure 1:**
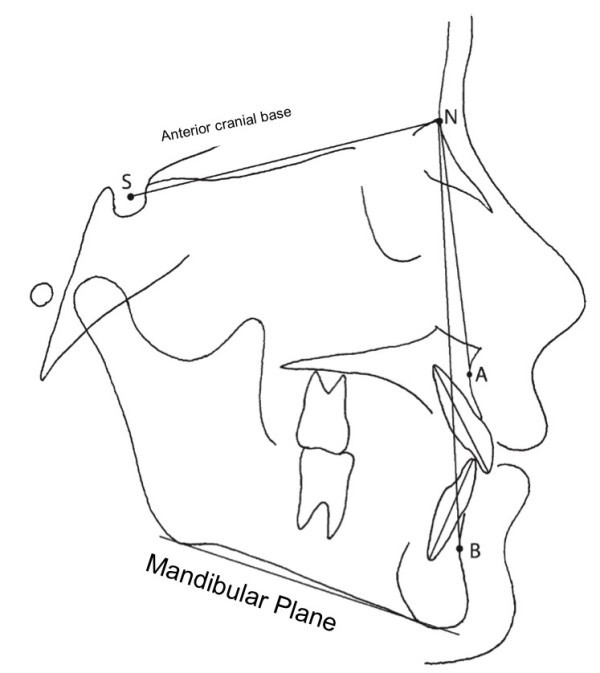
**Cephalometric references used in the study.**

**Figure 2:**
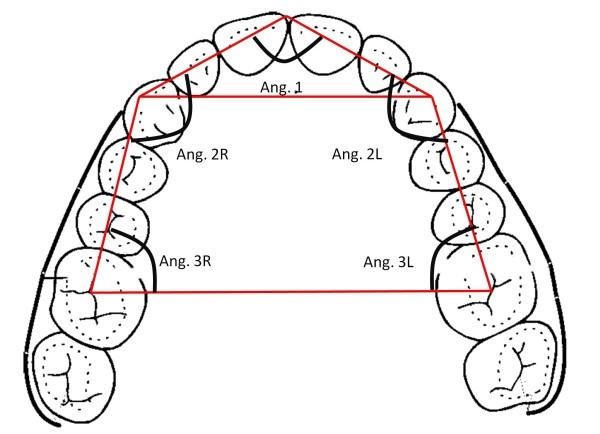
**Method and parameters for the shape analysis of the maxilla jaw.**

**Figure 3:**
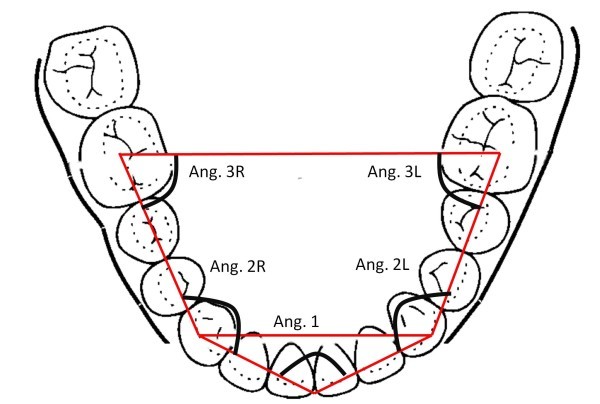
**Method and parameters for the shape analysis of the mandible jaw.**

A specific software allowed us to draw a pentagon inscribed inside the arches. A vertex of the pentagon was placed between the two central incisors; two other vertices lie on the cusp of the canines, and the other two were placed at the centre of first molars. Internal angles of the pentagon were measured. The ratio between the intercanine distance and the intermolar distance was calculated (Figures 1, 2, and 3).

The analysis was performed on both dental arches, the upper and lower, in an independent manner.

#### Method error

The described method was experimentally tested to avoid bias from the magnification of the images. Ten couples of plaster models and their digital photographs were measured. Firstly, linear and angular measurements were taken using a clear simmetro supported on the plaster models, than the same measures were calculated on digital images and compared with the first ones. The magnitude of the method error was calculated using Dahlberg’s formula [13]. The method error for the angular measurements ranged from 0.1° to 0.3° for each angle.

The measurements on the same couples of the digital images were re-measured after 4 weeks, and the new method error was calculated using Dahlberg’s formula [13]. The method error was within 0.4 mm for linear measures and within 0.5° for the angular measurements.

### Statistical analysis

Descriptive statistics, including the mean and SD, were calculated for all measurements. Pearson correlation was used to analyze the relationship between the arch form and the facial vertical dimension.

The differences between the three groups were identified through an analysis of variance (ANOVA ) followed by *post hoc* Bonferroni tests.

## Results

Table 1 shows descriptive statistics for each measurement. Table 2 shows the arch form measurements in the three SN-MP angle groups (low, medium, and high). All measurements except the ratio between the intercanine and intermolar distance are reported as angular values. The significance of each calculated value in the three groups is shown in Table 3. The value of significance is set at *P* < 0.05. In the maxilla, the angular values found in the three groups did not show differences that are statistically significant, with the exception of the angle value Ang. 1. The ratio between the intercanine and intermolar distance in the upper arch showed a *P* value < 0.05 indicating that the group with low angle had an intercanine diameter proportionately greater than groups with medium and high-angle SN-MP. These last two groups progressively showed a lower value. *Post hoc* Bonferroni test confirmed the significance of differences of ratio between the intercanine and intermolar distance between the three groups.

**Table 1:** **Descriptive analysis**

	Ang. 1	Ang. 2R	Ang. 2L	Ang. 3R	Ang. 3L	Intercanine distance	Intermolar distance	Intercanine-intermolar distance ratio	SN-MP
Mandible									
Number	73	73	73	73	73	73	73	73	73
Mean	135.2	131.6	131.4	70.6	71.1	25.1	38.9	0.6	32.7
Median	135.0	132.0	131.0	70.0	72.0	23.3	37.2	0.6	32.0
Standard deviation	9.9	6.5	7.9	4.7	4.7	6.9	9.2	0.05	7.3
Minimum	108.0	115.0	112.0	61.0	52.0	15.6	24.9	0.5	19.0
Maximum	166.0	148.0	164.0	85.0	81.0	51.2	67.1	0.8	50.0
Maxilla									
Number	73	73	73	73	73	73	73	73	73
Mean	125.7	131.0	131.1	75.5	76.1	30.4	41.7	0.7	32.7
Median	124.0	131.0	131.0	76.0	77.0	28.7	39.5	0.7	32.0
Standard deviation	9.7	7.4	7.9	4.3	4.5	8.1	9.7	0.06	7.3
Minimum	110.0	98.0	107.0	66.0	64.0	17.3	27.3	0.6	19.0
Maximum	152.0	147.0	149.0	86.0	86.0	56.1	66.1	0.9	50.0

**Table 2:** **Results for low, medium, and high SN-MP angle**

	Low SN-MP angle (<30.5°), ***n*** = 28			Medium SN-MP angle (30.5° to 35.5°), ***n*** = 19			High SN-MP angle (>35.5°), ***n*** = 26		
	Mean	Median	DS	Mean	Median	DS	Mean	Median	DS
Maxilla									
Ang. 1	129.4	129.5	10.2	123.5	123	8.9	123.5	122	8.9
Ang. 2R	130.6	131.5	5.6	130.9	131	5.7	133.4	134.0	5.9
Ang. 2L	130.3	130.5	6.0	132.7	132.0	6.7	133.0	133.0	6.3
Ang. 3R	75.2	76.0	4.5	75.3	75	4.1	75.9	76	4.4
Ang. 3L	75.9	76.5	4.5	76.8	78	4.3	75.6	76	4.8
Intercanine and intermolar distance ratio	0.7	0.7	0.0	0.7	0.7	0.0	0.7	0.7	0.0
Mandible									
Ang. 1	132.9	134	9.3	132.9	132	5.7	139.3	138.5	11.8
Ang. 2R	133.6	132	7.1	132.4	133	5.3	128.9	130.5	5.7
Ang. 2L	132.9	132	6.2	132.3	132	5.3	129.0	128.5	5.7
Ang. 3R	69.5	69	4.8	71.1	70	3.4	71.3	70	5.1
Ang. 3L	71	70.5	4.6	71.2	72	4.2	71.2	72	5.4
Intercanine and intermolar distance ratio	0.6	0.6	0.0	0.6	0.6	0.0	0.6	0.6	0.0

**Table 3:** **Variance analysis**

		Sum of squares	***df***	Mean square	***F***	Sig.
Mandible						
Ang. 1	Between groups	675.944	2	337.972	3.682	0.030
	Within groups	6,424.741	70	91.782		
	Total	7,100.685	72			
Ang. 2R	Between groups	301.021	2	150.510	3.910	0.025
	Within groups	2,694.240	70	38.489		
	Total	2,995.260	72			
Ang. 2L	Between groups	223.268	2	111.634	1.839	0.167
	Within groups	4,249.745	70	60.711		
	Total	4,473.014	72			
Ang. 3R	Between groups	51.033	2	25.516	1.144	0.324
	Within groups	1,560.638	70	22.295		
	Total	1,611.671	72			
Ang. 3L	Between groups	0.384	2	0.192	0.008	0.992
	Within groups	1,613.507	70	23.050		
	Total	1,613.890	72			
Intercanine and intermolar distance ratio	Between groups	0.002	2	0.001	0.310	0.734
	Within groups	0.199	70	0.003		
	Total	0.201	72			
Maxilla						
Ang. 1	Between groups	586.772	2	293.386	3.294	0.043
	Within groups	6,234.721	70	89.067		
	Total	6,821.493	72			
Ang. 2R	Between groups	122.683	2	61.342	1.846	0.200
	Within groups	2,325.837	70	33.226		
	Total	2,428.521	72			
Ang. 2L	Between groups	112.911	2	56.455	1.416	0.250
	Within groups	2,790.212	70	39.860		
	Total	2,903.123	72			
Ang. 3R	Between groups	7.383	2	3.692	0.193	0.820
	Within groups	1,336.781	70	19.097		
	Total	1,344.164	72			
Ang. 3L	Between groups	17.690	2	8.845	0.424	0.650
	Within groups	1,458.638	70	20.838		
	Total	1,476.329	72			
Intercanine and intermolar distance ratio	Between groups	0.061	2	0.031	9.461	0.000
	Within groups	0.227	70	0.003		
	Total	0.288	72			

Only the mandibular arch angular values Ang. 1 showed a statistical significance (*P* value < 0.05), while values Ang. 2R, Ang. 2L, Ang. 3R, and Ang. 3L were not significant and the ratio between the intercanine and intermolar distance was also not significant in the lower jaw. The angular value Ang.1 increased from the low- to high-angle groups. *Post hoc* Bonferroni test proved the significance of Ang. 1 between low- to high-angle groups.

Table 4 showed Pearson correlation of the relationship between the arch form and the facial vertical dimension. The angles that express the anterior arch form (Ang1, Ang2R, Ang2L) were correlated with the variation in facial vertical dimension. The value of *R* was low and the value of the coefficient of determination *R*^2^ did not explain more than about 10% of the variance. The strongest correlation was found between the vertical dimension and the distances ratio in the upper arch (−0.420). The value of negative sign indicated an inverse correlation, for which increasing vertical dimension decreased the value of the ratio, and then the arch appeared narrower in the intercanine area.

**Table 4:** **Pearson correlation**

	SN/MP		
	***R***	Sig.	***R*** ^2^
Maxilla			
Ang.1	−0.364^a^	0.002	0.132
Ang.2R	0.276^a^	0.018	0.076
Ang.2L	0.279^a^	0.017	0.077
Ang.3R	0.093	0.435	0.008
Ang.3L	−0.040	0.739	0.001
DR	−0.420^a^	0.000	0.176
Mandible			
Ang.1	−0.263^a^	0.024	0.069
Ang.2R	−0.344^a^	0.003	0.118
Ang.2L	−0.244^a^	0.038	0.059
Ang.3R	0.238^a^	0.042	0.056
Ang.3L	−0.077	0.518	0.005
DR	−0.004	0.976	0.000

^a^Significant.

## Discussion

Previous studies were focused on the evidence of variability of dental arch forms in skeletal class I patients. Forster [8] found decreased wideness of dental arch with increased SN-MP angles in subjects with skeletal class I, in both males and females. Our research was aimed to find out comparable results in skeletal class II patients (ANB > 5°) who represent the most prevalent malocclusion in Caucasians. As the untreated subjects were not recruited from a population sample but from a university dental clinic, some inherent bias might be possible.

Giuntini et al. [14] found that upper intermolar width was significantly smaller in class II malocclusion than class I malocclusion. Our study confirmed a significant deficiency in the upper intermolar width along with a significant negative posterior transverse interarch discrepancy in class II subjects when compared with class I subjects. Therefore, it appears that a transverse deficiency of maxillary arch is a typical finding in growing subjects with class II malocclusion also in the presence of upper molar rotation.

Result analysis showed a change in upper arch shape with an intercanine diameter proportionately smaller in patients with high angles and greater in patients with low angles (*P* < 0.05). The bigger the SN-MP angles were, the narrow is the form of the upper arches. Those results were in accordance with what was described for skeletal class I. Furthermore, the same correlation between the vertical dimension and the width of the maxillary arch was noticed, independently to sagittal discrepancy between the two jaws. Although the data from the present study showed an inverse trend between SN-MP angle and dental arch widths, the correlation was not very strong. It seems that the SN-MP angle might be only one of the contributing factors.

The mandibular and maxillary angular values were different between left and right. This discovery of an asymmetric tendency tempts one to ask if, perhaps, the pursuit of a symmetrical arch is not an affront to nature that guarantees a degree of relapse [15].

There was no statistically significant difference in mandibular arch forms between the three groups with the exception of the angle value Ang. 1. The increase of this value from low- to high-angle groups should be interpreted as the prevalence of ‘V’ shapes arch form in subjects with low angle and of ovoid arch forms in high-angle patients.

To obtain a correct archform, it is desirable to achieve more posttreatment stability [15]; therefore, the aim of most research on dental arch forms is to uncover if the preformed archwires fits all patients.

Nowadays, the use of nickel titanium preformed archwire, in association with straight wire techniques, is widespread. The risk is that the results are not stable because the technique and materials do not fit the patient anatomy.

Bhowmik et al. [16] found a difference among gender, showing that usually female archwires are smaller than that of the male ones. Furthermore, they found that all preformed archwires were larger than the true arch form of the investigated sample, leading to posttreatment instability.

In literature, there are many papers [16, 17] on this topic, but they all refer to class I populations. All of which lead to the conclusion that the preformed archwires do not fit for most of our patients, and their use can produce unfavorable side effects, such as excessive intercanine width. Mandibular intercanine and intermolar widths are accurate indexes of patient inherent muscular balance and, in most cases, dictate the limits of arch expansion in these areas during treatment.

This highlights the importance of using individualized archwires according to pretreatment arch form and width for each patient during orthodontic treatment. Since the wide variations in patient arches cannot be met by the few preformed archwire shapes and sizes available, the concept of individualization of archwires is strongly suggested. Furthermore, even between class I patients, there are some differences when mandibular growth direction is considered.

## Conclusions

Arch form is a unique expression of individual development because there are many small but significant variations in arch shapes. In this study, conclusions can be summarized as follows:

- The vertical growth patterns are correlated with the transverse growth of the upper arch in skeletal class II.
- This correlation is not very strong. It seems that the SN-MP angle might be only one of the contributing factors.
- Changes in upper arch shape with intercanine diameter proportionately smaller in patients with high angles and larger in low-angle patients are shown.
- As the form of dental arches is associated with vertical growth patterns, it would be desirable to use individualized arches for each patient respecting the characteristic of the arch form.

## Authors’ information

CG is an associate professor of Dentistry and Chairman of Postgraduate School in Orthodontics, Catholic University of Sacred Heart. BO is a visiting professor of Catholic University of Sacred Heart. RD is the Chief of Orthodontic Department of Catholic University of Sacred Heart. SS and ALG are postgraduate students in Orthodontics.
